# A 15-year-old male with embryonal rhabdomyosarcoma of the testis—a rare tumor with unusual presentation

**DOI:** 10.1093/jscr/rjaf166

**Published:** 2025-03-27

**Authors:** Wael A Abuisneneh, Batool Darraj, Nagham A Shalaldeh, Shahd Abdelhafez, Radwan Amro, Anwar Yousef Jabari

**Affiliations:** Faculty of Medicine, Palestine Polytechnic University, Hebron, Palestine; Urology Department, Yatta Hospital, Hebron, Palestine; Faculty of Medicine, Palestine Polytechnic University, Hebron, Palestine; Faculty of Medicine, Palestine Polytechnic University, Hebron, Palestine; Faculty of Medicine, Palestine Polytechnic University, Hebron, Palestine; Faculty of Medicine, Palestine Polytechnic University, Hebron, Palestine; Urology Department, Yatta Hospital, Hebron, Palestine; Faculty of Medicine, Palestine Polytechnic University, Hebron, Palestine

**Keywords:** rhabdomyosarcoma, testis, orchidectomy, incidental finding

## Abstract

Paratesticular embryonal rhabdomyosarcoma (ERMS) is a rare and aggressive mesenchymal tumor with only 24 reported cases in the literature. It is usually seen in children and adolescents presenting as a painless testicular mass. Hereby, we report a 15-year-old male patient who was diagnosed with testicular ERMS during exploratory surgery after testicular trauma. Diagnostic modalities include radiological imaging, histopathological examination, and immunohistochemical analysis. Treatment consists of radical inguinal orchiectomy followed by postoperative chemotherapy and radiotherapy, which has shown promising outcomes in managing this type of tumor.

## Introduction

Embryonal rhabdomyosarcoma (ERMS) of the testis, a type of soft tissue sarcoma, is an exceedingly rare and aggressive malignancy, representing ⁓3% of childhood cancers and 1% of adult cancers, with an incidence rate of 4.5 per 1 million individuals [1].

This type of sarcoma arises from mesenchymal cells and is more commonly found in the paratesticular region rather than the testis itself [2]. An ERMS presenting as a painful mass after trauma is an unusual presentation. The diagnosis is suspected through the combination of radiological imaging and histopathological examination. A comprehensive multimodal treatment plan, including surgical resection, radiotherapy, and chemotherapy, is essential for effective management [3, 4]. To date, only 23 cases have been reported in the literature [4, 5].

In this report, we describe a challenging case of a 15-year-old male who was initially misdiagnosed with an inguinal hernia after testicular trauma, emphasizing the diagnostic challenges of this rare cancer and the importance of considering ERMS as a differential diagnosis following trauma.

## Case presentation

A 15-year-old male patient presented to the emergency department with severe testicular pain and swelling post-trauma. It happened by accident at school, where his groin region hit the angle of the table. During the past 7 months, the patient noticed a left-sided painless scrotal swelling that slowly progressed in size. Since then he neglected it in a way that he didn’t even tell his parents or seek medical advice, thinking that it was a hernia. On physical examination, a painful, huge red mass was noted in the left scrotum without other significant findings. He’s a non-smoker, and he has no family history of similar conditions. Ultrasound was done and revealed a ruptured tunica albuginea in the left testicle with surrounding blood, but no other significant intratesticular abnormalities were identified.

The next step involved exploratory surgery to repair or remove the ruptured testicle based on the extent of the rupture. During surgery, the surgeon observed a grossly and abnormally shaped left testicle, prompting suspicion of malignancy due to the highly abnormal testicular architecture, which was unlikely to result from trauma. So an orchiectomy was performed on the left testicle. Post-operative imaging, including a positron emission tomography (PET) scan from the skull vertex to the toes, showed no suspicious focal uptake or lesions in the brain and no hypermetabolic lymph nodes in the cervical, mediastinal, axillary, or hilar regions. In the abdominal and pelvic regions, evidence of the previous left-sided orchiectomy was noted with focal fluorodeoxyglucose (FDG) uptake at the left spermatic cord, indicating post-operative changes. Blood tests, including serum glutamic pyruvic transaminase (SGPT), albumin, alkaline phosphatase, serum glutamic oxaloacetic transaminase (SGOT), blood urea nitrogen (BUN), calcium, direct bilirubin, FT3, FT4, TSH, and lactate dehydrogenase (LDH), were all within normal limits.

The macroscopic examination of the specimen showed a 15 × 11 × 10 cm mass with ruptured tunica vaginalis. Step sectioning revealed an ill-defined whitish mass with foci of necrosis occupying the whole testis.

**Figure 1:**
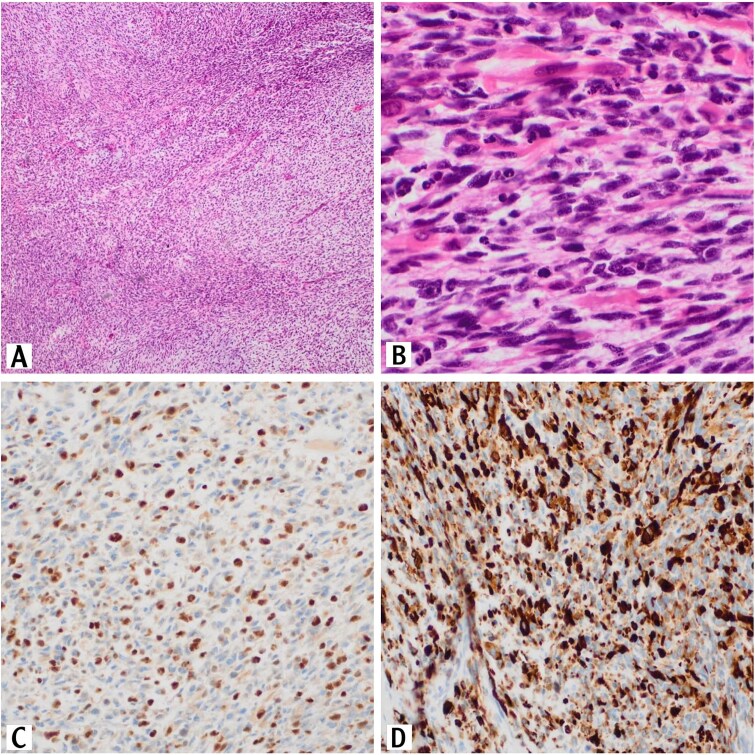
Sections of the testicular mass revealed proliferation of spindle cells that arranged in fascicles (H&E, 10×). These cells exhibit variable degree of nuclear pleomorphism with tumor necrosis and high mitotic activity (H&E, 10×) (A). Numerous rhabdomyoblasts are also present (H&E, 40×) (B). The tumor cells are positive for myogenin immunostain (20×) (C). The tumor cells are positive for desmin immunostain (20×)(D).

Histopathologic examination revealed a proliferation of spindle cells that are arranged in fascicles and numerous Rhabdomyoblasts are also present (Fig. 1A and B).

On Immunohistochemically, tumor cells showed positive staining for desmin and myogenin (Fig. 1C and D).

These pathology results were consistent with the diagnosis of para-testicular ERMS.

Following the initial surgery, the patient underwent a second operation to excise the tumor completely and is currently receiving chemotherapy and radiotherapy. Follow-up imaging and evaluations are planned to monitor for recurrence or metastasis.

## Discussion

This paper presents a case of a 15-year-old male who was diagnosed with testicular ERMS as an incidental finding during exploratory surgery after testicular trauma, and what makes this case even more special, a worthy-written one, is that the malignancy suspicion was settled on only when the testicle was seen by the surgeon’s eye during orchiectomy, whereas till this moment, everything was highly supportive of normal consecutive events following testicular trauma (including physical examination and primary ultrasonographic diagnostic measure).

ERMS of the testis is a rare and aggressive tumor, mostly seen in children and young adults [1, 6]. Whereas rhabdomyosarcoma (RMS) itself is the most common soft tissue sarcoma in pediatrics and adolescence, accounting for up to 50% of soft tissue sarcomas in this age group. In contrast, RMS is uncommon in adults, representing just 3% of soft tissue sarcomas [6]. RMS is a mesenchymal, high-malignant tumor with frequent recurrence. According to the WHO in 2013, RMS is categorized into four histological subtypes: alveolar, embryonal, pleomorphic, and spindle cell/sclerosing type. Alveolar RMS is characterized by rapid progression, early metastasis, and a higher mortality rate compared to ERMS [7]. It occurs in different places in the body, including the head and neck and the genitourinary system. Among these locations, primary testicular ERMS is exceptionally rare, with only 24 reported cases in the literature [4, 5].

Intrascrotal involvement of RMS can be classified into intratesticular and paratesticular forms. Primary intratesticular rhabdomyosarcomas (PITRMs) are extremely rare, comprising ˂1% of all testicular sarcomas, while para testicular RMS are relatively more common, constituting ⁓7% of intrascrotal RMS [2, 3].

Various factors have been linked to the development of testicular ERMS, such as cryptorchidism, trauma, advanced maternal age, paternal cigarette smoking, exposure to exogenous maternal estrogen, and X-ray exposure [8].

Clinically, it typically presents as a painless testicular mass that gradually increases in size over a relatively brief period. Painful masses have been reported in a few cases. Apart from that, the symptoms are not specific [3].

ERMS is diagnosed using a variety of methods, including radiological imaging (ultrasound, computed tomography (CT), and magnetic resonance imaging (MRI), histopathology, and immunohistochemistry staining (IHC). Ultrasound is the most usable imaging technique for examining intratesticular mass and, in combination with abdominal CT, for differentiating between intratesticular and paratesticular intrascrotal masses [1, 3, 4].

Histologically, ERMS exhibits primitive oval to spindle cells with little cytoplasm. Small, spherical, blue cells can be seen in some places. These cells, which are referred to as “tadpole,” “strap,” and “spider” cells and are thought to be indicative of rhabdomyoblastic differentiation [4, 7].

Immunohistochemically, muscular markers that have been used include anti-desmin, actin, and myoglobin antibodies, with desmin being the most frequently used marker, which exhibits diffuse staining [7].

Treatment of ERMS is challenging due to the limited availability of cases and the lack of extensive exploratory trials. However, according to several studies, the most effective treatment for ERMS is radical inguinal orchiectomy followed by postoperative chemotherapy and radiotherapy. The recommended chemotherapy drugs are vincristine, actinomycin-D, and cyclophosphamide (VAC) [1, 6].

This case underscores the critical need to consider malignancy in similar presentations, as delayed diagnosis can lead to disease progression and poorer treatment outcomes.

## Conclusion

ERMS of the testis is an extremely rare, aggressive tumor, with only 24 cases having been reported in the literature worldwide. As a result, limited research has been conducted on this type of tumor, indicating the need for further exploration and understanding. The rarity of this condition, along with its potential to mimic other testicular issues, poses a diagnostic challenge. The incidental finding of this malignancy post-trauma can lead to early intervention. Early diagnosis of these tumors, in addition to aggressive surgical treatment in combination with chemotherapy, reduces the incidence of local recurrence and enhances the overall prognosis.

## Data Availability

The data used to support the findings of this study are included in the article.
